# 3D printing of dynamic covalent polymer network with on-demand geometric and mechanical reprogrammability

**DOI:** 10.1038/s41467-023-37085-9

**Published:** 2023-03-10

**Authors:** Zizheng Fang, Yunpeng Shi, Hongfeng Mu, Runzhi Lu, Jingjun Wu, Tao Xie

**Affiliations:** 1 ZJU-Hangzhou Global Scientific and Technological Innovation Center, No. 733, Jianshe San Road, Xiaoshan District, Hangzhou, Zhejiang, 311200 China; 2 State Key Laboratory of Chemical Engineering, College of Chemical and Biological Engineering, Zhejiang University, 866 Yuhangtang Road, Hangzhou, 310030 P.R. China; 3 Ningbo Innovation Center, Zhejiang University, 1 Qianhu South Road, Ningbo, 315807 P.R. China

**Keywords:** Polymers, Polymers, Mechanical properties

## Abstract

Delicate geometries and suitable mechanical properties are essential for device applications of polymer materials. 3D printing offers unprecedented versatility, but the geometries and mechanical properties are typically fixed after printing. Here, we report a 3D photo-printable dynamic covalent network that can undergo two independently controllable bond exchange reactions, allowing reprogramming the geometry and mechanical properties after printing. Specifically, the network is designed to contain hindered urea bonds and pendant hydroxyl groups. The homolytic exchange between hindered urea bonds allows reconfiguring the printed shape without affecting the network topology and mechanical properties. Under different conditions, the hindered urea bonds are transformed into urethane bonds via exchange reactions with hydroxyl groups, which permits tailoring of the mechanical properties. The freedom to reprogram the shape and properties in an on-demand fashion offers the opportunity to produce multiple 3D printed products from one single printing step.

## Abstract (web summary)

3D printing allows preparation of versatile polymer materials for device applications, but properties are often fixed following printing. Here, the authors report a photo-printable dynamic covalent network that allows modification of properties following printing.

## Introduction

Complex geometric structures with tailorable mechanical properties are vital for multifunctional devices. Recent advances in 3D printing have led to unprecedented freedom in accessing arbitrary geometries^1–8^. For synthetic polymers, the unlimited topological connectivity between the same set of monomer units offers countless opportunities for tuning their mechanical properties^9,10^. Harnessing the topological diversity of polymers for 3D printing is, however, hindered by the often stringent printing requirements. Amongst all 3D printing technologies, digital light processing (DLP) stands out in its high precision and speed^3–8^. Typically, its liquid precursor is photo-cured into a crosslinked solid. The photo-curing and flowing requirements of the liquid resin severely constrain the material options. In addition, the formation of a crosslinked network is such that the printed object is difficult to change, in terms of both the macroscopic shape and microscopic network topology. This “one print to one product” scenario greatly limits the scope of 3D printing.

Dynamic covalent chemistry, by virtue of the reversible bond forming and breaking, offers unusual opportunities to design polymer networks with adaptive functions including self-healing and reprocessing^11–21^. Of particular relevance here is that dynamic bond exchange also allows shape reconfiguration of a crosslinked network via plasticity^22–24^. Herein, plasticity refers to permanent shape reconfiguration associated with macroscopic deformation with no entropy gains due to the ability of dynamic networks to undergo bond exchange to relax the deformed chains to their thermodynamically stable configurations^22^. The unique adaptability arises from topological network rearrangement. Importantly, the network topology is unaltered before and after the rearrangement, that is, the mechanical properties remain unchanged^25,26^. On the contrary, recent work shows that a dynamic network can also be designed to switch to different topologies. In such a so-called topology isomerizable network (TIN)^27,28^, the non-uniform chain segmental distribution in the original network, referred to as topological heterogeneity, drives the network transformation towards topologically more homogeneous states by bond exchange. Critically, the different topological states determine the macroscopic properties. Thus, the TIN mechanism allows tailoring mechanical properties in a programmable manner.

Incorporating dynamic chemistries including Diels-Alder^29^, disulfide^30^, imine^31^, *β*-hydroxyl esters^32^, thioester-anhydride^33^, thiourethane^34^, and boronate ester^35^ in 3D printing has led to geometrically complex devices capable of self-healing and reprocessing. However, the network designs are such that the printed shapes and mechanical properties cannot be reprogrammed after printing. We deduce that, if a network can be designed to undergo independent plasticity-based shape reconfiguration^22–24^ and topology isomerization^27,28^, the “one print to one product” can potentially become “one print to unlimited products”. Although the idea is theoretically achievable at the network chemistry level, meeting 3D printing requirements is a major challenge that should be addressed. In particular, existing examples of TIN^27,28^ rely on topological heterogeneity as the entropic driving force for isomerization, namely the redistribution of the same covalent bond connections. This necessitates the use of macromonomers as the network building units, making it difficult for DLP printing due to the low concentration of polymerizable moieties and the high melt viscosity.

In this work, we demonstrate a DLP printable dynamic covalent polymer network with independent shape reconfiguration and topology isomerization capabilities. Notably, its topology isomerization is facilitated by the formation of new covalent bonds with higher bond energy, that is, the isomerization is enthalpy driven instead of entropy driven. With such a chemistry design, we illustrate that a 3D printed object can be reprogrammed into multiple objects of different geometric shapes and mechanical properties, extending markedly the scope of 3D printing. We should emphasize that our concept is fundamentally different from the well-known post-curing adjustment of material properties/shape of 3D printed parts^32,36–38^, for which the non-fully cured intermediate states are unstable given the unreacted monomer(s) present. In addition, the post-curing approach does not allow independent adjustment of properties and shapes.

## Results

### Network design and rearrangement mechanisms

**Fig. 1:**
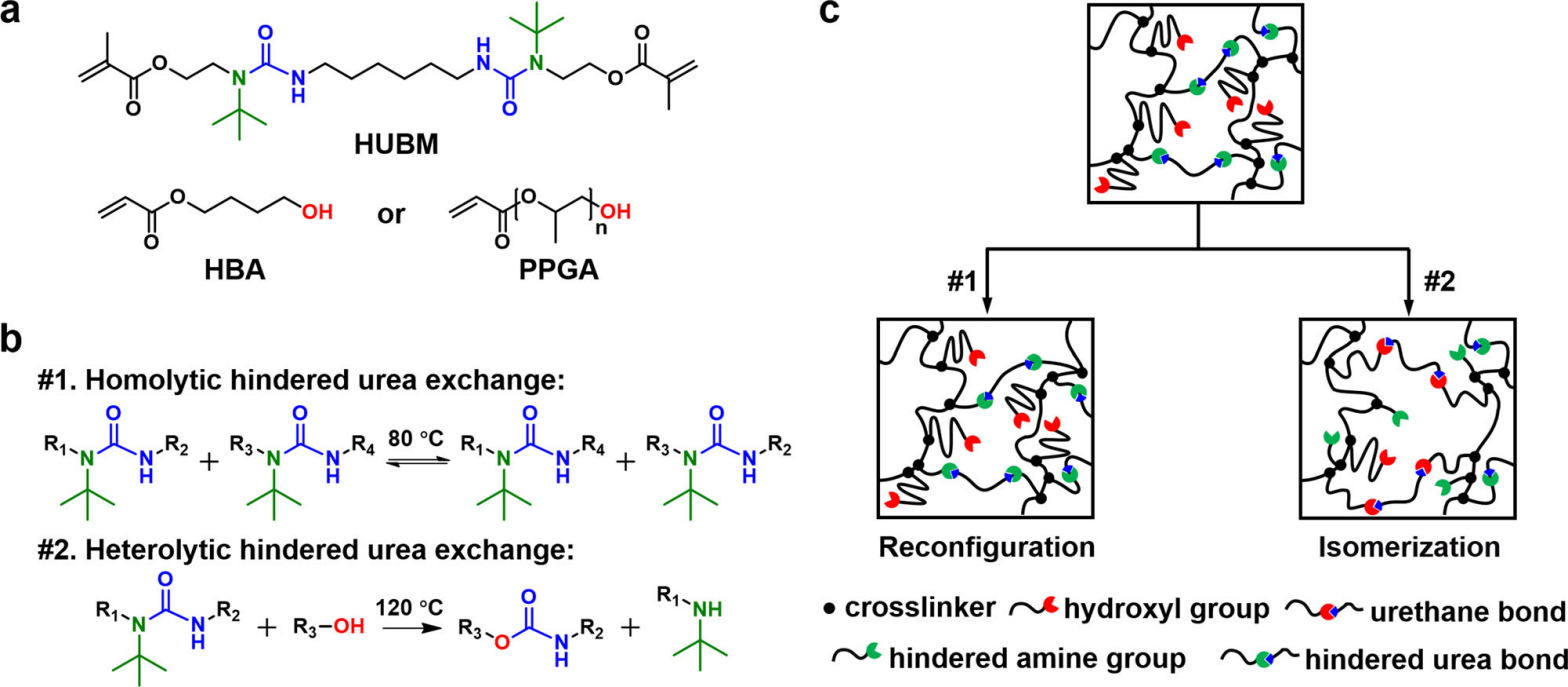
Molecular design of the 3D printable dynamic network. **a** Monomer precursors. **b** The homolytic and heterolytic exchange reactions at different temperatures. **c** Schematic illustration of two distinct network rearrangement mechanisms.

The DLP liquid printing precursor consists of a hindered urea containing bismethacrylate (HUBM) and a hydroxyl-terminated acrylate (Fig. 1a). Their molar ratio is kept at 1:2 in order to maintain equal molarity between the hindered urea bonds and the hydroxyl group. Here, the HUBM is synthesized with its structure verified in Supplementary Figs.1–3. For the hydroxyl-terminated acrylate, either 4-hydroxybutyl acrylate (HBA, *M*_n_ = 144) or poly(propylene glycol) acrylate (PPGA, *M*_n_ = 475) is employed. As revealed later in this work, switching between these two acrylates of different chain lengths provides the freedom to tune the mechanical properties of the resulting network. The networks are synthesized by light-mediated free-radical polymerization in the presence of a photo-initiator (Irgacure 819, 3 wt% of the total precursor). For such networks, two distinct rearrangement mechanisms can occur conceptually. At a relatively low temperature (80 °C), the dynamic hindered urea bonds are activated for homolytic bond exchange (Fig. 1b)^39–41^, which allows reprogramming of the macroscopic shape via plasticity (Fig. 1c). At a sufficiently high temperature (120 °C), the hindered urea bonds react with the hydroxyl groups to yield energetically more stable urethane bonds and hindered amine groups (Fig. 1b)^42^. This latter heterolytic exchange chemistry results in enthalpy-based topology isomerization (Fig. 1c). With drastically different bond formation (i.e. urethane), the mechanical properties are consequently altered. Despite the different bond formations, the molecular formula of the network, which is a giant molecule with infinite molecular weight, remains unchanged. The process is therefore referred to as topological isomerization. We should note that, in the above process, the homolytic bond exchange is reversible while the heterolytic one is irreversible. However, this heterolytic exchange can be interrupted at any point by lowering the temperature. This allows access to any topological states between the original network and the fully isomerized network. These kinetically trapped intermediate topological states correspond to the continuous evolution of materials of different mechanical properties. Following the above principles, the geometry and mechanical properties of the network polymer can be independently reprogrammed.

**Fig. 2:**
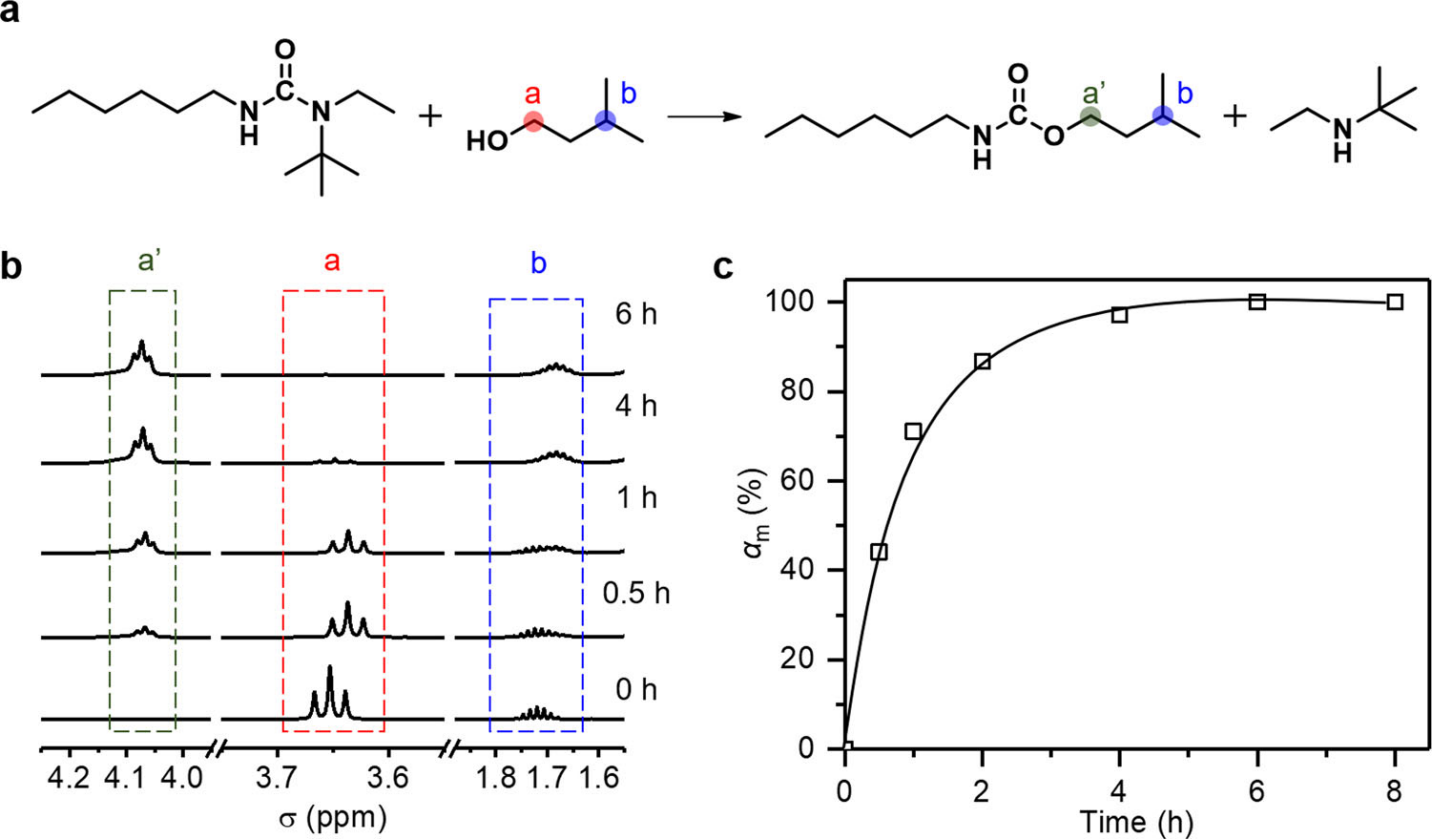
Model compound study of n-hexyl-n-tert-butylethyl-urea and 3-methyl−1-butanol. **a** Heterolytic exchange chemistry that transforms hindered urea into urethane. **b** ^1^H NMR monitoring of the model reaction. **c** Kinetics of the bond conversion at 120 °C.

It is generally known that the homolytic exchange of hindered urea bonds occurs at relatively low temperatures (below 80 °C)^39–41^, but the heterolytic transformation from a hindered urea to a urethane at higher temperatures is not well established quantitatively for polymer networks. Accordingly, a model compound experiment is designed to quantify the latter reaction. A hindered urea compound n-hexyl-n-tert-butylethyl-urea (Fig. 2a) is synthesized and its ^1^H MNR spectrum is presented in Supplementary Figs. 4–6. It is then reacted with an equal molar amount of a primary alcohol (3-methyl-1-butanol) at a relatively high temperature of 120 °C. The kinetics of the exchange reaction between a hindered urea and a primary alcohol (Fig. 2a) is investigated using ^1^H MNR analysis. Specifically, the peak change in the alcohol (from *a* to *a'*) is quantitatively monitored using peak *b* as the internal normalization standard (Fig. 2a, b). Here, the chemical shift of peak *b* decreases with the reaction, which is due to the change in its surrounding chemical environment. The reaction conversion *α*_m_ is calculated as the ratio between the normalized integrated area of peak *a'* and the total normalized integrated area of *a* and *a'* (Fig. 2b). The results (Fig. 2c) suggest that the hindered urea is fully converted into the urethane in ~4 h at 120 °C, notably under a catalyst-free condition. In principle, a concurrent transesterification exchange reaction between the ester bond from the acrylates and the hydroxyl groups may also occur in the process, especially when a hindered amine (a transesterification catalyst) is present^42–47^. To investigate this possibility, two model compound experiments were conducted. In the first experiment, a monomer containing an ester group and a hindered amine is reacted with a primary alcohol at 120 °C. The result (Supplementary Fig. 7) suggests that transesterification indeed occurs, reaching a reaction conversion of around 35% after 4 h. In the second model experiment, a monomer containing an ester group and a hindered urea (Supplementary Figs. 8–10) is reacted with primary alcohol at 120 °C. From NMR and QTOF analysis (Supplementary Figs. 11 and 12), there is no detectable transesterification product even after 6 h of reaction. The result indicates that the hydroxyl group reacts dominantly with the hindered ureas rather than the esters. Together, these two model compound studies verify that, with the coexistence of a hindered urea and ester bond, transesterification is suppressed and the hydroxyl group reacts dominantly with the hindered urea and transforms it into urethane (Supplementary Figs. 11 and 12).

**Fig. 3:**
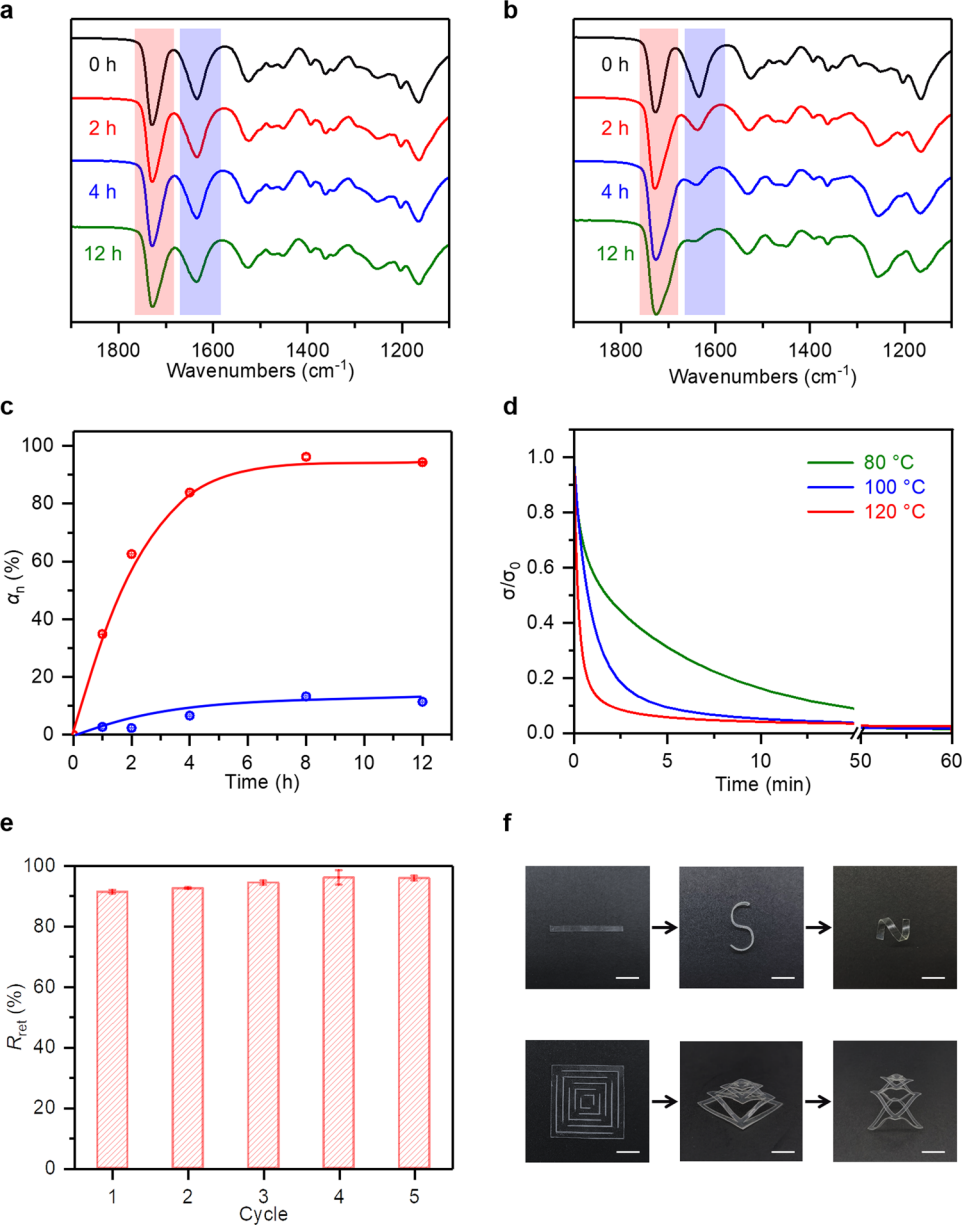
The exchange reactions in a polymer network (HUBM-co-HBA). **a**, **b** Time evolutions of the Fourier transform infrared (FTIR) spectra at 80 and 120 °C, respectively. **c** The conversions from hindered urea to urethane (*α*_n_) at 80 and 120 °C. **d** Iso-strain stress relaxation curves (strain is fixed at 10%). **e** Calculated shape retention after multiple cycles of shape reconfiguration (80 °C, 60 min). **f** Visual demonstration of the shape reprogrammability. Scale bar, 10 mm.

We next use the network obtained by curing between HUBM and HBA as a model to probe homolytic and heterolytic exchange chemistries using infrared (IR) analysis. After postcuring in a UV chamber, the sample HUBM-co-HBA is fully cured as indicated by its FTIR spectrum (Supplementary Fig. 13), showing that the characteristic acrylate peak^39^ at 809 cm^−1^ has vanished. The light-cured sample is thermally annealed at 80 °C and the change in its carbonyl peaks (1580–1780 cm^−1^) is monitored. Figure 3a shows that, for the initial network (0 h), the carbonyl peaks for the hindered urea and the esters appear at 1635 cm^−1^ and 1725 cm^−1^, respectively^48^. As the annealing proceeds for up to 12 h, these two peaks do not seem to alter drastically. We note here that the carbonyl peak associated with urethane, if produced, is expected to overlap with the ester peaks. Nevertheless, the fact that the integrated area of the hindered urea peak does not change notably suggests that hindered urea bonds are not converted into urethane bonds. In contrast, when the annealing is conducted at 120 °C (Fig. 3b), the integrated area of hindered urea peak undergoes a rather notable reduction and the integrated area of peak at 1725 cm^−1^ is broadened. This implies the conversion from hindered urea to urethane bonds. The comparison between Fig. 3a and Fig. 3b suggests that the transformation from hindered urea to urethane is minimal at 80 °C, but becomes significant at 120 °C. This conversion from hindered urea to urethane can be quantitatively calculated from the infrared analysis (see experimental section in Supplementary Information). Accordingly, Fig. 3c shows that the conversion is only around 10% even after annealing at 80 °C for 12 h. By comparison, the conversion increases drastically at 120 °C, reaching a high plateau value of about 90% at around 8 h.

### Shape and mechanical reprogrammability

Although the heterolytic exchange is largely suppressed at 80 °C, the homolytic exchange is expected to occur at this temperature. With our design intent, this would lead to shape reconfiguration capability via plasticity, which should be reflected in the stress relaxation behavior^22,39^. Specifically, iso-strain stress relaxation due to the bond exchange allows the initially deformed chains in their non-equilibrium configurations to return to their equilibrium configurations, which correspond to permanent shape reconfiguration at the macroscopic level. Accordingly, iso-strain stress relaxation experiments are conducted using the cured network. The results (Fig. 3d) show that the sample can relax stress almost completely at 80 °C in 60 min. Increasing the temperature accelerates the stress relaxation, with complete relaxation achieved in 5 min at 120 °C. Within these time and temperature scales (80 °C for 60 min or 120 °C for 5 min), the heterolytic exchange is expected to be quite minimal (<5%) based on the results in Fig. 3c. This implies that the homolytic and heterolytic exchange can be independently controlled. Such a controlling mechanism arises from the different temperature dependence of the two exchange reactions, namely Arrhenius dependency. The stress relaxation due to homolytic exchange is directly related to shape reconfigurability quantified as the shape retention ratio *R*_ret_ = *ε*_final_/*ε*_initial_, where *ε*_initial_ and *ε*_final_ denote the initially applied strain and the final strain after the stress relaxation followed by stress removal, respectively^22,39^. Figure 3e shows that *R*_ret_ is around 90% in the first cycle of shape reconfiguration (80 °C, 60 min) and it maintains a high level even after five cycles. Figure 3f demonstrates more directly the shape reprogrammability, with the initial sample being altered to drastically different shapes with repeated reconfiguration.

As the design intent, annealing at 120 °C for a sufficiently long time triggers the heterolytic exchange for topology isomerization, which can be used to reprogram the (thermo-)mechanical properties. Supplementary Fig. 14 shows that, after annealing for 12 h, the glass transition temperature (*T*_g_) changes from 99 °C to 83 °C. The modulus and breaking stress, however, are largely unchanged accompanied by a variation from 1.16 ± 0.06 GPa to 1.12 ± 0.06 GPa, and from 55.5 ± 6.3 MPa to 53.1 ± 5.2 MPa. The results suggest that, although the properties can indeed be reprogrammed, their achievable range is not significant. We deduce that this is due to the selection of HBA as the comonomer, which is too similar to the tert-butylamine produced during the isomerization, both in terms of molecular weight and structure.

**Fig. 4:**
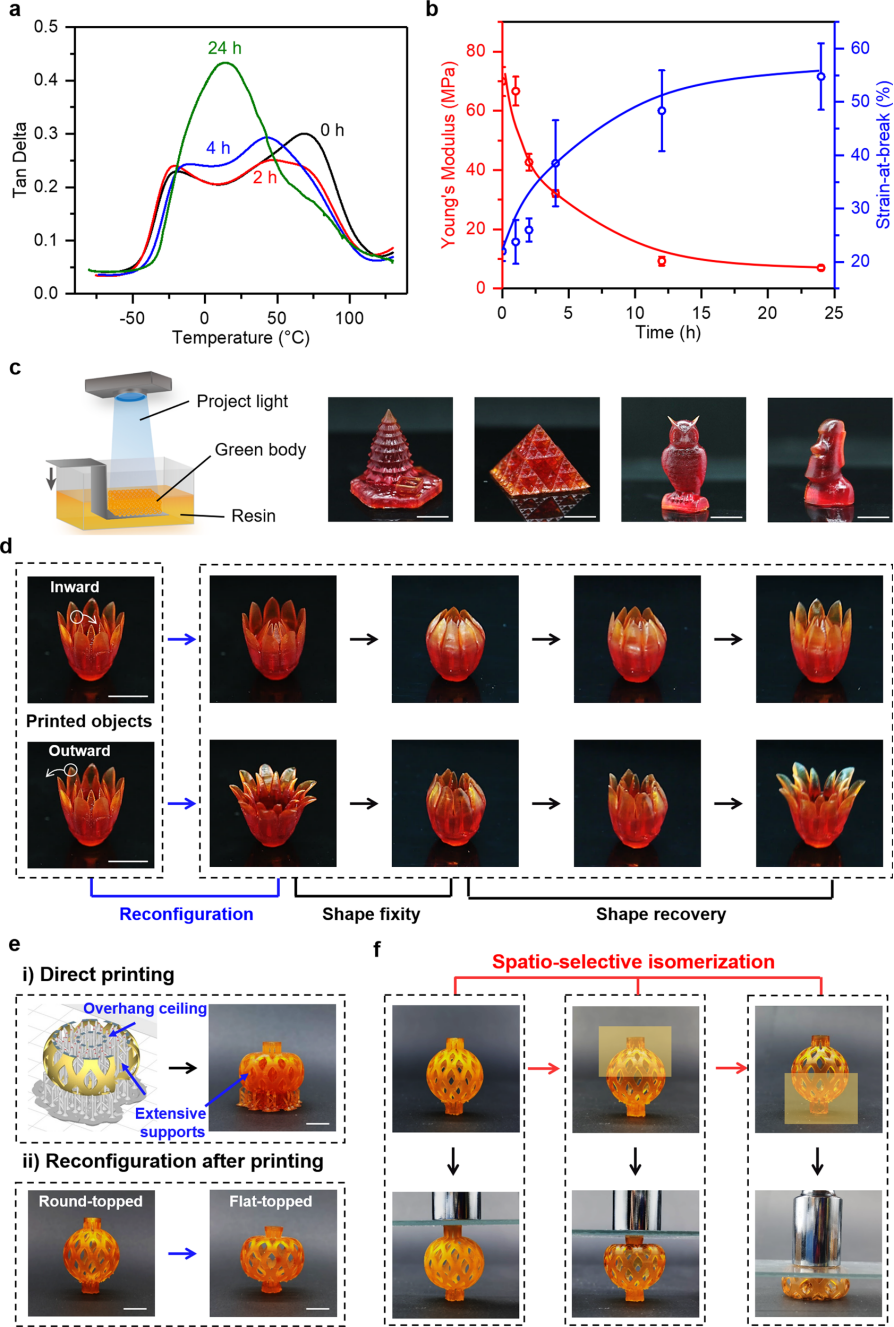
Shape and mechanical reprogrammability for digital light 3D printed HUBM-co-PPGA. **a** Evolution of thermal transitions upon annealing at 120 °C. **b** Evolution of mechanical properties upon annealing at 120 °C. **c** Schematic illustration of the printing process and photos of the 3D printed objects. **d** Permanent shape reconfiguration with the printed flower petals being permanently deformed inward or outward (80 °C, 60 min) and their corresponding shape memory behaviors with shape fixing and recovery at −40 °C and 60 °C. **e** The construction of the flat-topped lantern structure via (i) direct printing and (ii) reconfiguration after printing. **f** Spatio-selective isomerization of the printed lantern structure and the corresponding compression behaviors under the same load. (The yellow regions were immersed in a heated oil bath at a temperature of 120 °C for 6 h). Scale bar, 10 mm.

In order to expand the tunable range so as to enlarge the benefit of topological programmability, we switch to PPGA with a much longer poly(propylene glycol) as the comonomer. For the HUBM-co-PPGA network, its stress relaxation at 80 °C is conducted under the same condition. Its value of *R*_ret_ (Supplementary Fig. 15) is lower than that of the HUBM-co-HBA network (Fig. 3e). This is due to the lower mass content of dynamic motifs (HUBM) for the HUBM-co-PPGA network in order to maintain equal molarity between the hindered urea bonds and the hydroxyl group in the formulation. The transformation from hindered urea to urethane at 120 °C is also confirmed by infrared analysis (Supplementary Fig. 16). The as-synthesized sample exhibits two *T*_g_s at −22 °C and 69 °C (Fig. 4a), most likely due to the different reactivities of PPGA and HUBM (acrylate versus methacrylate) and the phase separation upon photo-curing due to the incompatibility between the polymerized PPGA and HUBM rich phases. Both *T*_*g*_s for the HUBM-co-PPGA network are lower than that of HUBM-co-HBA. This is due to its lower mass fraction of HUBM in the formulation and that the *T*_g_ of the homopolymer of PPGA is lower than that of the homopolymer of HBA (Supplementary Fig. 17). Upon isomerization at 120 °C, both *T*_g_s change notably (Fig. 4a), ultimately merging into a single broad *T*_g_ at 14 °C after annealing for 24 h. The change of *T*_g_s is presumably because of the formation of hydrogen bonds in the polyether-based urethane and vanishing of hydrogen bonds in the hindered urea motifs in HUBM. Correspondingly, its modulus at 25 °C declines ten times from 70 MPa to 7 MPa whereas the strain-at-break increases from 20–55% (Fig. 4b). The modulus decrease upon isomerization arises from the conversion from two phases (with one rigid phase) to one soft phase. This is also responsible for the increase of strain-at-break, in addition to the contribution of the increase in the chain length between crosslinking points (Supplementary Tab. 1). The average molecular weight between crosslinks *M*_c_ is calculated from the swelling ratio based on the Flory-Rehner equation, where the value of *M*_c_ is increased from 186 ± 14 kg/mol to 271 ± 4 kg/mol after isomerization.

### Digital light 3D printing

The above study sets up the stage to make 3D printed products with reprogrammable shapes and properties using the formulation with PPGA as the comonomer. Figure 4c demonstrates that a diverse set of complex 3D structures can be successfully printed using a top-down DLP setup. To demonstrate the shape reprogrammability, the printed flower (Fig. 4d) is repeatedly and sequentially reconfigured (80 °C, 1 h under an external deformation force) to yield multiple complex shapes. Three points are worth noting here. First, all these shapes are thermodynamically stable permanent shapes as heating to 60 °C (above the *T*_g_s) does not induce any shape change. Second, the newly produced permanent shapes can be further manipulated with its glass transitions as the shape memory transition, that is, they can fix the temporary shape and recover to their permanent shape (Fig. 4d, with quantitative shape memory cycles in Supplementary Fig. 18). Third, the derivative shapes with overhanging features are difficult to 3D print directly unless extensive structural support is also printed, which would require cumbersome manual removal. To better illustrate the significance of the shape reprogramming, a representative example of a lantern structure with a flat top is set as the target printing object (top row in Fig. 4e). The flat top, more like overhang ceiling, requires extensive supports during printing as shown by the digital 2D profile diagram. These supports that reside in the interior of the lantern are difficult to remove because they are nearly impossible to access manually. With our method, however, one can print a round-topped lantern without having to use any support inside the lantern (bottom row in Fig. 4e). Afterwards, the round-top can be reconfigured into a flat-top (80 °C, 1 h). The comparison in Fig. 4e illustrates one of the advantages of shape reprogramming after printing versus direct printing.

Another advantage lies in its unusual flexibility in multi-material integration, which is one of the unique features of 3D printing over existing processing methods. Although light curing based multi-material printing are known practices, there are notable drawbacks/inefficiencies. For instance, commercial multi-material printers by PolyJet rely on selectively spaying different curable resins onto each printing layer followed by UV curing^49^. It requires multiple UV resins and the printer is very costly. Another approach^50^ relies on switching precursor baths that contain different UV curable precursors during printing. Having to resort to multiple printing precursors for multi-material printing leads to printing inefficiencies and complexity in printing control. In contrast, our material concept opens up a way for multi-material integration using a single printing precursor and the most common DLP printing setup. As illustrated above, annealing at 120 °C for 24 h under a stress-free condition triggers the heterolytic exchange for topological isomerization and the printed structure can be transformed into a softer material without changing the geometric shape. Uniquely, spatio-selective isomerization is possible by exposing a printed sample to a non-uniform temperature field. This is illustrated in Fig. 4f, showing that the as-printed lantern structure is hardly compressed under a load of 10 kg due to the rigidity. With the upper part immersed in a heated oil bath at 120 °C, it is converted into a mechanically non-uniform construct. The upper part is softened due to isomerization whereas the lower part remains rigid. Consequently, only the upper part is compressible under the same load. Of course, if the lower part is subsequently thermally treated in the same way, the entire object becomes uniformly soft. Thus, our approach of mechanical reprogramming after printing allows unusual freedom for multi-material integration.

In addition, the homolytic and heterolytic exchange chemistries can be used to reprogram the shape and properties in a synergistic way. Specifically, after a printed shape is reprogrammed to a new shape at 80 °C, its mechanical properties can be further manipulated by isomerization at 120 °C (Supplementary Fig. 19). We should emphasize that our printed material at any intermediate topological states are near-fully cured networks (with gel fractions above 90%, see Supplementary Tab. 2). And the mechanical properties change is nearly consistent with the results tested before as curing in a UV chamber (Supplementary Tab. 3). In addition, the corresponding mechanical properties at these states have been measured after storing the samples at room temperature for 2 weeks. The results show that the samples with different storage times have identical properties, suggesting that any intermediate topological states correspond to networks that are stable over time at room temperature (Supplementary Tab. 4).

## Discussion

In summary, we illustrate an unusual versatility for 3D printing, namely “one print to multiple products” instead of “one print to one product” for currently known 3D printing. This freedom arises from the unique dynamic covalent chemistry design that provides the printed network with two embedded bond exchange mechanisms. The homolytic exchange of hindered urea bonds allows reconfiguring the geometric shape. The heterolytic exchange that converts the hindered urea bonds to urethane bonds permits reprogramming the mechanical properties. The two mechanisms can operate independently and synergetically for on-demand manipulation of 3D printed products, importantly even by ordinary customers. Here, FDM 3D printing employs polymer filaments and can be used in regular households. DLP 3D printing has a much higher printing resolution, but unfortunately uses UV curable liquids. This makes DLP unsuitable for regular households. With our technology, one can order a DLP printed product made in a printing shop. Depending on the need, end customers can alter the shape and mechanical properties on-demand without having to handle hazardous liquid. Besides that, expanding the underlying material principle to other dynamic covalent chemistries can broaden the range of material tunability, with vast potential for flexible manufacturing of geometrically complex multi-functional devices.

## Methods

### Materials

2-(Tert-butylamino)ethyl methacrylate (TBEMA, 98%), n-tert-butylethylamine (TBEA, 98%), hexyl isocyanate (98%), and bis(2,4,6-trimethylbenzoyl)-phenylphosphineoxide (Irgacure 819) were purchased from TCI (Tokyo Chemical Industry, Japan). Poly(propylene glycol) acrylate (PPGA, *M*_n_ = 475) was received from Sigma-Aldrich (USA). Hexamethylene diisocyanate (HDI, 99%), 4-hydroxybutyl acrylate (HBA, 97%) and Sudan III were obtained from Aladdin (China). 3-Methyl-1-butanol (99.5%) was provided by Macklin (China). All chemicals were used as received.

### Digital light 3D printing

The liquid printing precursor was prepared by mixing HUBM and PPGA at a molar ratio of 1:2. 3% Photoinitiators (Irgacure 819) and 0.05% photoabsorber (Sudan III) were subsequently introduced. After being stirred to a transparent state, the obtained precursor mixture was displaced in dark before use. Its viscosity was 127 mPa/s, tested using a digital viscometer (NDJ-5S, Shanghai HengPing Instrument and meter Factory, China). Printing was conducted using a bottom-up 3D printer with a commercial projector (DELL 1609WX, USA., 10 mW cm^−2^). The exposure time for each layer was set as 20 s. The printed sample was post-cured in a UV chamber (IntelliRay 600 Flood UV, Uvitron International, USA, 955 mW cm^−2^, 265~700 nm) with an exposure time of 120 s (Supplementary Fig. 20).

### Characterization

All the ^1^H NMR and ^13^C NMR spectra were measured using Bruker, Avance III (USA) with CDCl_3_ as the solvent. FTIR spectra were conducted using a Nicolet iS50 FTIR spectrometer (Thermo Fisher, USA) with a scanning range from 4000 to 400 cm^−1^. Quadrupole time-of-flight (QTOF) mass spectrometry was recorded with a Bruker Ultraflex mass spectrometer. Gel fractions were tested by immersing the samples in tetrahydrofuran (THF) and a minimum of three specimens were tested for each sample. Stress relaxation experiments were performed using DMA Q800 (TA instruments, Germany) in a “stress relaxation” mode with an iso-strain value set at 10%. In multiple stress relaxation cycles, the true cross-section area of the initial sample will diminish due to the cumulative elongation of the sample. To obtain a precise value of *R*_ret_ after multiple cycles, the sample was priorly stretched and subsequently relaxed for different cycles before it was tested using DMA with the same procedure (80 °C, 60 min). Glass transition temperatures (*T*_g_) and shape memory performance were tested using the same equipment in a “tension, multifrequency, strain” mode (1 Hz, 0.2% strain, heating rate: 3 °C min^−1^) and a “tension, control force” mode, respectively. The calculation of the shape fixity ratio (*R*_f_) and the shape recovery ratio (*R*_r_) can be found in our previous works^22,39^. Mechanical testing was performed at room temperature using a mechanical tester (BW91272, Zwick/Roell, Germany, load cell: 1 kN) at a speed of 100 mm min^−1^. The samples were cut into a standard dumbbell shape (20×2×0.4 mm^3^) with a laser cutter (Trotec Speedy100R, Germany) before the tests. The printed samples for mechanical testing were also designed as a dumbbell shape. At least five specimens were tested for each sample.

## Supplementary information

Supplementary Information

## Data Availability

The data that support the findings of this study are available from the corresponding author on request.
