# Supplementary material for: 3D printing of dynamic covalent polymer network with on-demand geometric and mechanical reprogrammability
Source: Nat Commun. 2023 Mar 10;14:1313. doi: 10.1038/s41467-023-37085-9 (PMC10006071; doi:10.1038/s41467-023-37085-9)
Supplement: Supplementary file 1 — Supplementary Information [file 41467_2023_37085_MOESM1_ESM.pdf]

Supplementary Information

3D Printing of Dynamic Covalent Polymer Network with on-Demand Geometric and Mechanical Reprogrammability

Zizheng Fang[1,2], Yunpeng Shi[2], Hongfeng Mu[2], Runzhi Lu[2], Jingjun Wu[2,3*], Tao Xie[2*]

[1]ZJU-Hangzhou Global Scientific and Technological Innovation Center, No. 733, Jianshe San Road, Xiaoshan District, Hangzhou, Zhejiang, China, 311200
[2]State Key Laboratory of Chemical Engineering, College of Chemical and Biological Engineering, Zhejiang University, 866 Yuhangtang Road, Hangzhou, 310030, P.R. China
[3]Ningbo Innovation Center, Zhejiang University, 1 Qianhu South Road, Ningbo, 315807, P.R. China

*Correspondence: jingjunwu@zju.edu.cn; taoxie@zju.edu.cn

This PDF file includes:
Supplementary Table 1 to 4.
Supplementary Figure 1 to 20.

Additional experimental descriptions

Synthesis of hindered urea containing bismethacrylate (HUBM). HUBM was synthesized as follows. 2-(Tert-butylamino)ethyl methacrylate (3.7 g, 0.02 mol) was added into a three-necked flask with a set temperature of 50 °C, followed by the dropwise addition of a stoichiometric amount of hexamethylene diisocyanate (1.68 g, 0.01 mol). After reaction under magnetic stirring for 5 hours, the product as a viscous liquid was obtained without further purification (yield: 99%). The structure was confirmed by ^{1}H NMR, ^{13}C NMR, FTIR, and QTOF analysis (Supplementary Figs. 1-3). The QTOF analysis exhibited m/z of 561.3625, which agreed well with the theoretical molecular weight (m/z: 561.3623) calculated for ($[C_{28}H_{50}N_4O_6]^+Na)^+$.

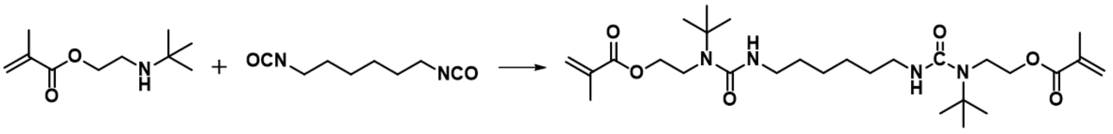

Model compound experiment of n-hexyl-n-tert-butylethyl-urea and 3-methyl-1-butanol. A monomer containing monofunctional hindered urea n-hexyl-n-tert-butylethyl-urea was obtained with the addition of an equimolar of n-tert-butylethylamine (1.01 g, 0.01 mol) and hexyl isocyanate (1.27 g, 0.01 mol) following the same procedure for HUBM (50 °C for 5 hours). The product as a liquid was obtained without further purification (yield: 99%). The structure was confirmed by ^{1}H NMR, ^{13}C NMR, FTIR, and QTOF analysis (Supplementary Figs. 4-6). The QTOF analysis exhibited m/z of 251.2093, which agreed well with the theoretical molecular weight (m/z: 251.2094) calculated for ($[C_{13}H_{28}N_2O]^+Na)^+$.

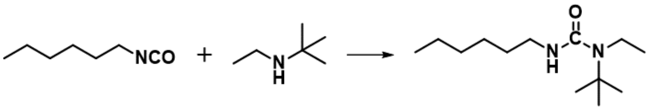

In the model compound experiment, a stoichiometric amount of n-hexyl-n-tert-butylethyl-urea (2.28 g, 0.01 mol) and 3-methyl-1-butanol (0.88 g, 0.01 mol) were mixed and allowed to react at 120 °C. The reaction conversion α_m was quantified by ^{1}H NMR analysis. Specifically, the α_m value was calculated as the ratio between the

integrated area of peak *a′* (3.7 ppm) and the total integrated area of *a* (3.9 ppm) and *a′* with all the peaks normalized using *b* (1.7 ppm) as the internal standard. All the results were normalized for calculation.

Model compound experiment of TBEMA and 3-methyl-1-butanol. A stoichiometric amount of 2-(tert-butylamino)ethyl methacrylate (1.85 g, 0.01 mol) and 3-methyl-1-butanol (0.88 g, 0.01 mol) were mixed and allowed to react at 120 °C. The reaction conversion α_1 was quantified by ^{1}H NMR analysis. Specifically, the α_1 value was calculated as the ratio between the integrated area of peak *a′* (2.75 ppm) and the total integrated area of *a* (2.87 ppm) and *a′* with all the peaks normalized using *b* (6.1 ppm) as the internal standard. All the results were normalized for calculation.

Model compound experiment of (2-(tert-butyl)-hexyl-urea)ethyl methacrylate and 3-methyl-1-butanol. (2-(Tert-butyl)-hexyl-urea)ethyl methacrylate was obtained with the addition of an equimolar of 2-(tert-butylamino)ethyl methacrylate (1.85 g, 0.02 mol) and hexyl isocyanate (1.27 g, 0.01 mol) following the same procedure for HUBM (50 °C for 5 hours). The product as a liquid was obtained without further purification (yield: 99%). The structure was confirmed by ^{1}H NMR, ^{13}C NMR, FTIR, and QTOF analysis (Supplementary Figs. 8-10). The QTOF analysis exhibited m/z of 335.2306, which agreed well with the theoretical molecular weight (m/z: 335.2305) calculated for ($[C_{17}H_{32}N_2O_3]^+Na)^+$.

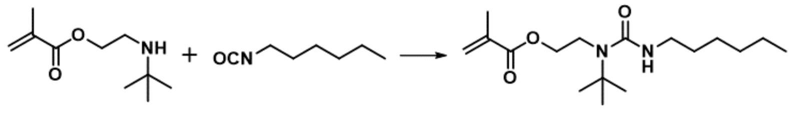

In the model compound experiment, a stoichiometric amount of (2-(tert-butyl)-hexyl-urea)ethyl methacrylate (3.12 g, 0.01 mol) and 3-methyl-1-butanol (0.88 g, 0.01 mol)were mixed and allowed to react at 120 °C. The reaction conversion α_2 was quantified by ^{1}H NMR analysis. Specifically, the α_2 value was calculated as the ratio between the integrated area of peak *a′* (4.27 ppm) and the total integrated area of *a* (4.2 ppm) and *a′* with all the peaks normalized using *b* (6.1 ppm) as the internal standard.

All the results were normalized for calculation.

Measurement of hindered urea bond conversion in polymer networks. The samples were annealed at a given temperature (80 °C or 120 °C) for different durations. The conversion of the hindered urea was measured by Fourier transform infrared (FTIR) spectrometer (Nicolet 5700 infrared spectrometer) by monitoring the integrated peak area of hindered urea group ($A_{\mathrm{hindered\ urea}}$, 1635 cm^{-1}) and urethane group (A_{urethane}, 1725 cm^{-1}). The conversion α_{n} was calculated as follows:

$$\alpha_n = 1 - \frac{(A_{hindered\ urea}/(A_{hindered\ urea}+A_{urethane}))_t}{(A_{hindered\ urea}/(A_{hindered\ urea}+A_{urethane}))_{t=0}} \quad (1)$$

All the results were normalized for calculation.

Swelling properties and the calculations of the average molecular weight between crosslinks M_{c}. The swelling experiments were conducted by immersing the samples in tetrahydrofuran (THF) and a minimum of three specimens were tested for each sample. The swelling degree Q is calculated from the following equation:

$$Q = \frac{\mathrm{m}_s - m_0}{\mathrm{m}_0} \quad (2)$$

where m_{s} and m_0 are corresponding to the weight of the swollen sample and dried sample.

According to the Flory-Rehner equation[1], the average molecular weight between crosslinks M_{c} can be calculated as follows:

$$\frac{1}{M_c} = \frac{2}{M_n} - \frac{\frac{\mathrm{V}_P}{V_l}[\ln(1-\mathrm{V}_K)+\mathrm{V}_K+\chi_{PK}V_K^2]}{\left(\mathrm{V}_K^{\frac{1}{3}}-\mathrm{V}_K/2\right)} \quad (3)$$

where V_{P} and V_{l} are corresponding to the specific volume of the polymer and molar volume of the solvent, M_{n} is the average molecular weight of the linear polymer before crosslinking, χ_{PK} is the Flory interaction parameter (selected as 0.46)[2]. V_{K} represents the volume fractions of the polymer, which can be calculated as follows:

$$\mathrm{V}_K = \frac{1}{1+Q} \quad (4)$$

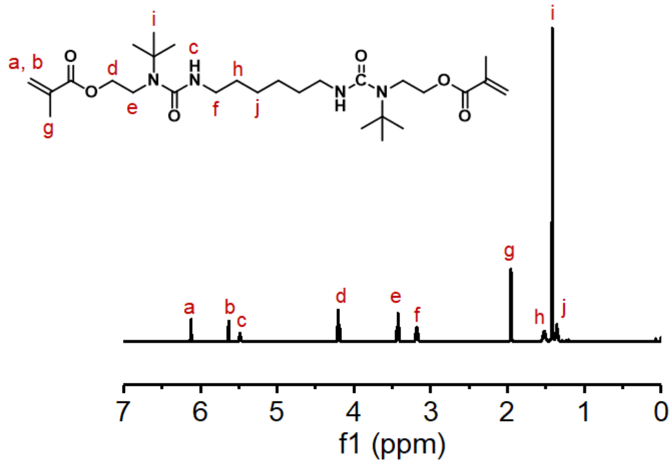

Supplementary Figure 1 ^{1}H NMR spectrum of HUBM. ^{1}H NMR (500 MHz, $CDCl_3$, δ): 6.12 (s, 1H, (a)), 5.63 (p, J=1.7 Hz, 1H, (b)), 5.46 (t, J=5.5 Hz, 1H, (c)), 4.20 (t, J=7.0 Hz, 2H, (d)), 3.43 (t, J=7.0 Hz, 2H, (e)), 3.18 (td, J=7.3, 5.3 Hz, 2H, (f)), 1.96 (t, J=1.5 Hz, 3H, (g)), 1.53 (q, J=7.0 Hz, 2H, (h)), 1.42 (s, 9H, (i)), 1.40-1.32 (m, 2H, (j)).

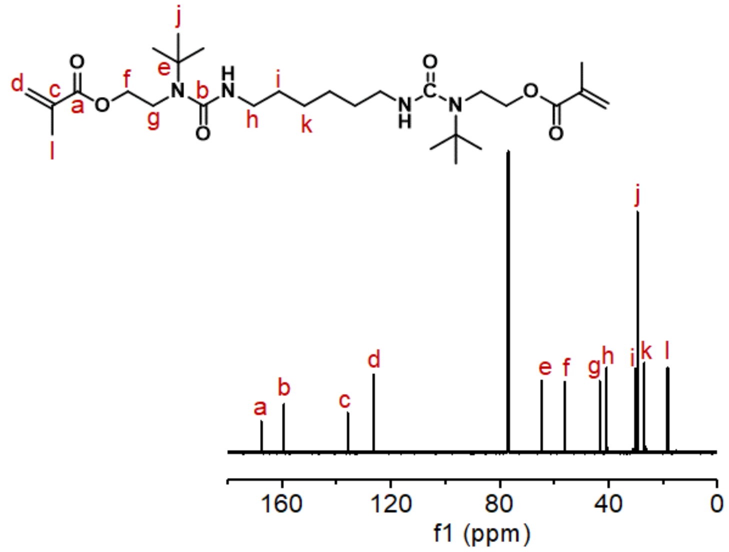

Supplementary Figure 2 ^{13}C NMR spectrum of HUBM. ^{13}C NMR (151 MHz, $CDCl_3$, δ): 167.68, 159.34, 135.86, 126.37, 64.72, 55.94, 43.31, 40.69, 30.12, 29.42, 26.80, 18.28.

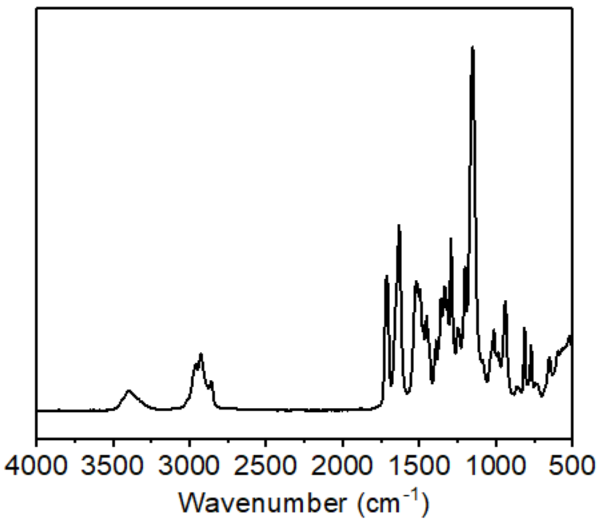

Supplementary Figure 3 FTIR spectrum of HUBM. The peak at 2264 cm^{-1} that corresponds to the stretching vibration of the isocyanate group completely disappears, indicating the successful synthesis of target products.

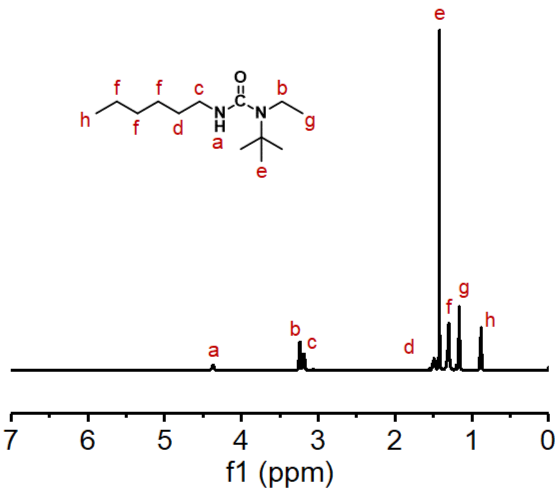

Supplementary Figure 4 ^{1}H NMR spectrum of n-hexyl-n-tert-butylethyl-urea. ^{1}H NMR (500 MHz, $CDCl_3$, δ): 4.37 (t, J=5.2 Hz, 1H, (a)), 3.28-3.14 (m, 4H, (b)(c)), 1.57-1.43 (m, 2H, (d)), 1.42 (s, 9H, (e)), 1.37-1.26 (m, 6H, (f)), 1.16 (t, J=7.2 Hz, 3H, (g)), 0.92-0.85 (m, 3H, (h)).

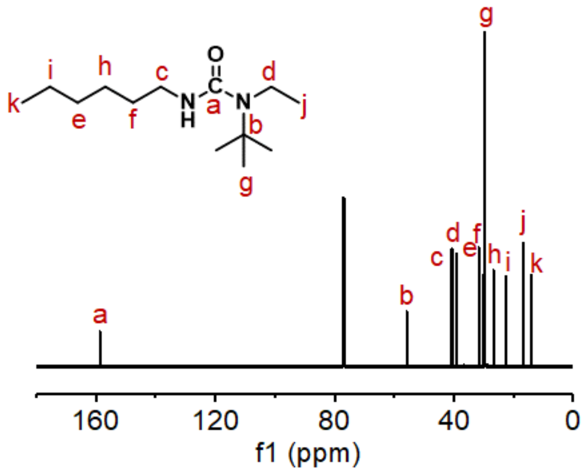

Supplementary Figure 5 ^{13}C NMR spectrum of n-hexyl-n-tert-butylethyl-urea. ^{13}C NMR (151 MHz, $CDCl_3$, δ): 158.69, 55.81, 40.65, 39.08, 31.56, 30.29, 29.67, 26.74, 22.58, 16.59, 13.97.

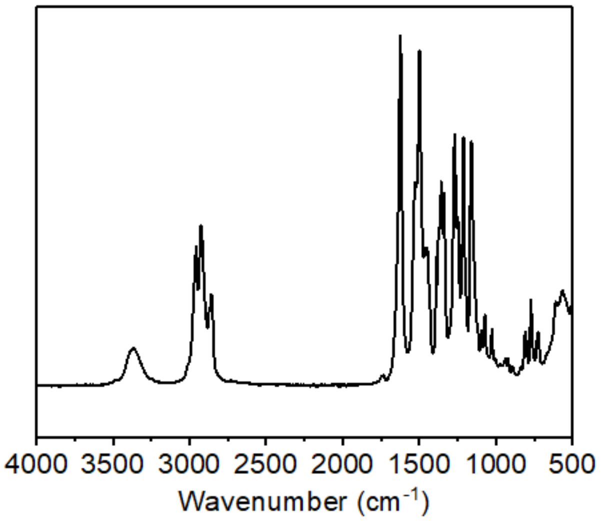

Supplementary Figure 6 FTIR spectrum of n-hexyl-n-tert-butylethyl-urea. The peak at 2264 cm^{-1} that corresponds to the stretching vibration of the isocyanate group completely disappears, indicating the successful synthesis of target products. The peak at 1635 cm^{-1} corresponds to the formation of the hindered urea bond.

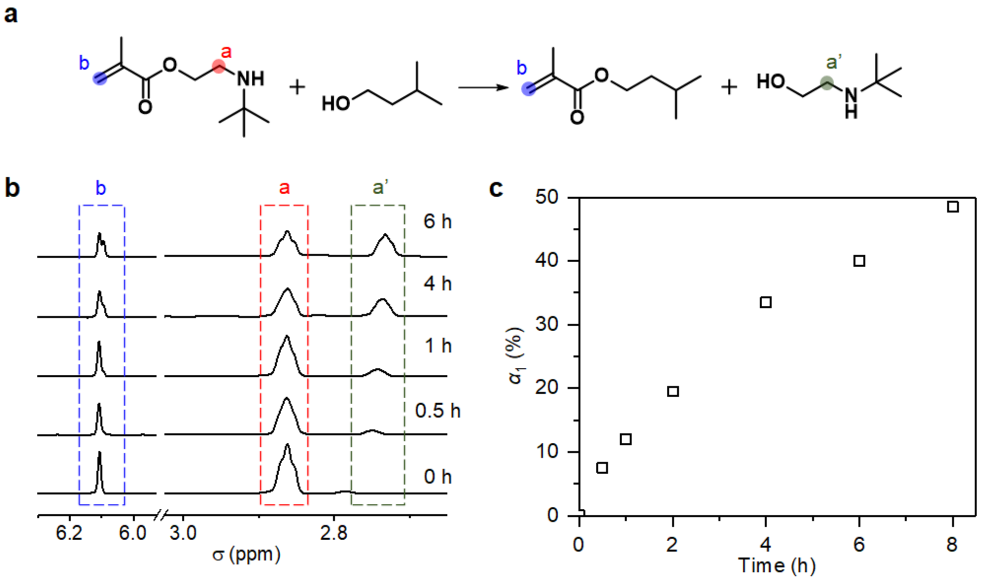

Supplementary Figure 7 Model compound study of TBEMA and 3-methyl-1-butanol. a Transesterification of the ester bond and the hydroxyl groups. **b** ^{1}H NMR monitoring of the model reaction. **c** Kinetics of the bond conversion at 120 °C.

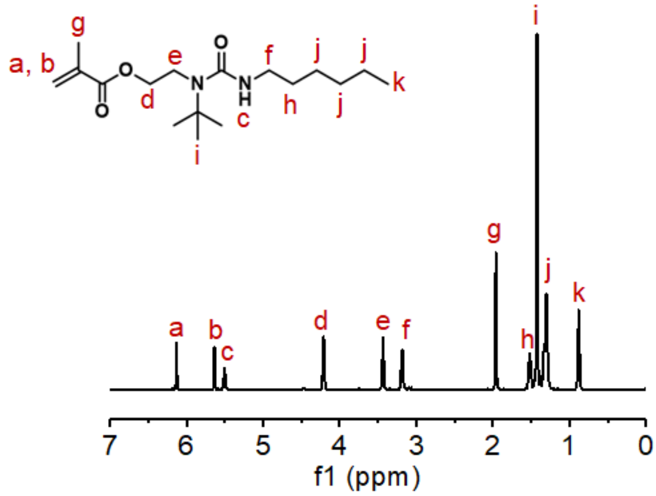

Supplementary Figure 8 ^{1}H NMR spectrum of (2-(tert-butyl)-hexyl-urea)ethyl methacrylate. ^{1}H NMR (500 MHz, $CDCl_3$, δ): 6.13 (s, 1H, (a)), 5.64 (s, 1H, (b)), 5.48 (d, J=6.2 Hz, 1H, (c)), 4.21 (t, J=7.1 Hz, 2H, (d)), 3.43 (t, J=7.2 Hz, 2H, (e)), 3.18 (t, J=7.0 Hz, 2H, (f)), 1.96 (s, 3H, (g)), 1.51 (q, J=7.5 Hz, 2H, (h)), 1.39 (s, 9H, (i)), 1.31 (d, J=15.9 Hz, 6H, (j)), 0.87 (d, J=7.0 Hz, 3H, (k)).

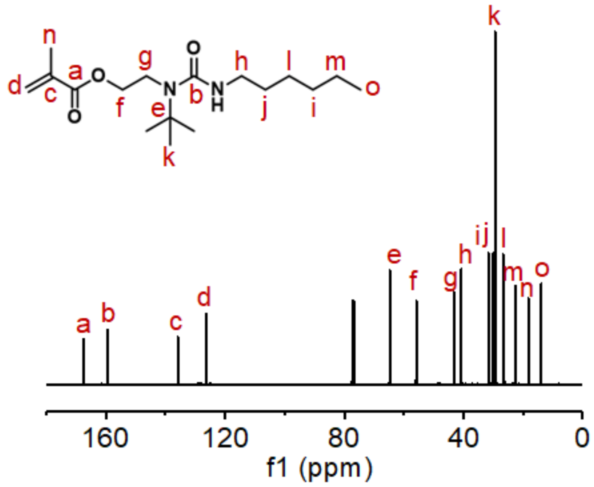

Supplementary Figure 9 ^{13}C NMR spectrum of (2-(tert-butyl)-hexyl-urea)ethyl methacrylate. ^{13}C NMR (151 MHz, $CDCl_3$, δ): 167.60, 159.30, 135.82, 126.26, 64.65, 55.86, 43.26, 40.77, 31.54, 30.01, 29.35, 26.72, 22.55, 18.20, 13.95.

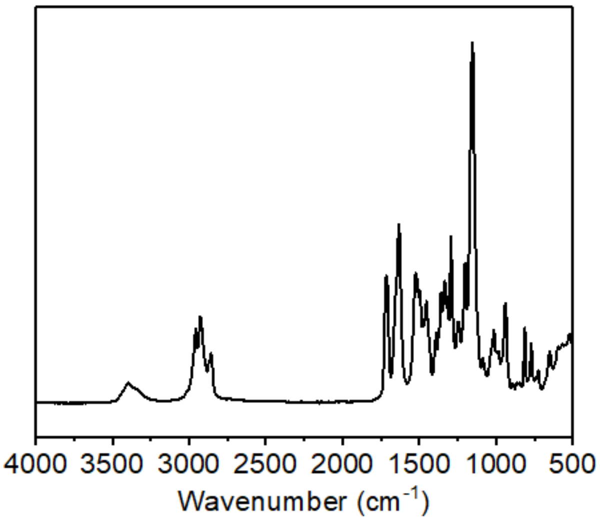

Supplementary Figure 10 FTIR spectrum of (2-(tert-butyl)-hexyl-urea)ethyl methacrylate. The peak at 2264 cm^{-1} that corresponds to the stretching vibration of the isocyanate group completely disappears, indicating the successful synthesis of target products.

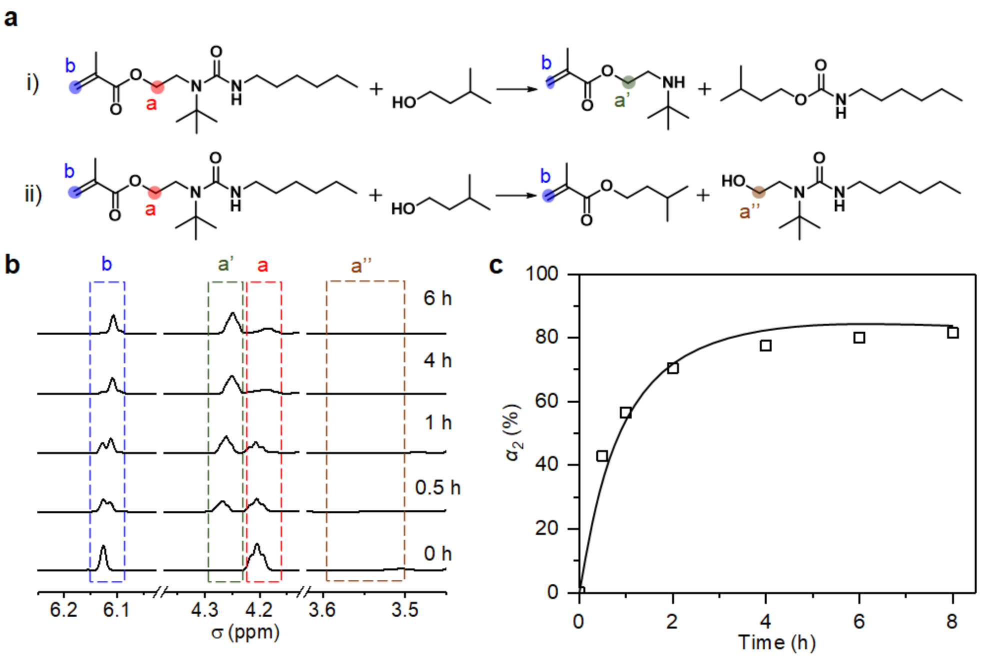

Supplementary Figure 11 Model compound study of (2-(tert-butyl)-hexyl-urea)ethyl methacrylate and 3-methyl-1-butanol. a The possible existence of two mechanisms. i) Heterolytic exchange chemistry that transforms hindered urea into urethane. ii) Transesterification. **b** ^{1}H NMR monitoring of the model reaction. The peak a″ (3.55 ppm) that corresponds to the transesterification products cannot be detected, indicating that the transesterification is negligible with the presence of a hindered urea and a primary alcohol. **c** Kinetics of the bond conversion at 120 °C.

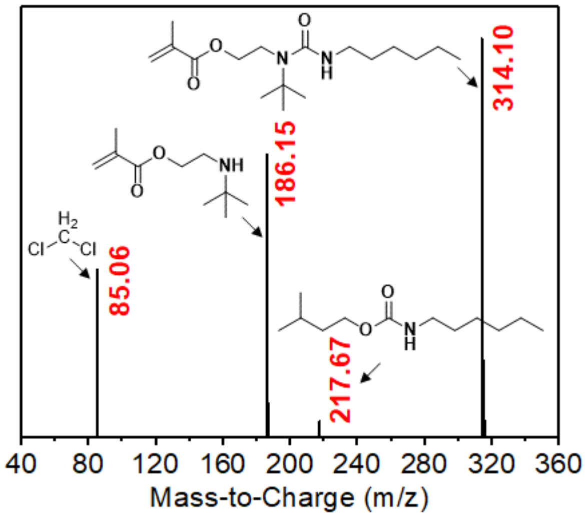

Supplementary Figure 12 QTOF analysis of the products of the model compound experiment in Supplementary Figure 11 (reaction time: 8 hours). The transesterification products are absent in the QTOF analysis, also suggesting that the model compound experiment in Supplementary Figure 11 follows the heterolytic exchange chemistry that transforms hindered urea into urethane.

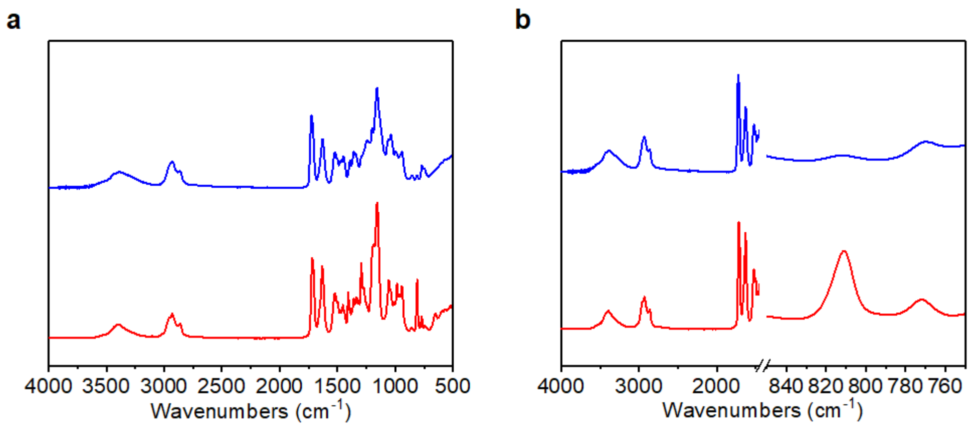

Supplementary Figure 13 FTIR spectrums of the precursor (containing HUBM and HBA, red line) and the photocured network HUBM-co-HBA (blue line). a The full spectrums range from 4000 cm^{-1} to 500 cm^{-1}. **b** The spectrums at a given range from 4000 cm^{-1} to 1450 cm^{-1}, and from 850 cm^{-1} to 750 cm^{-1}.

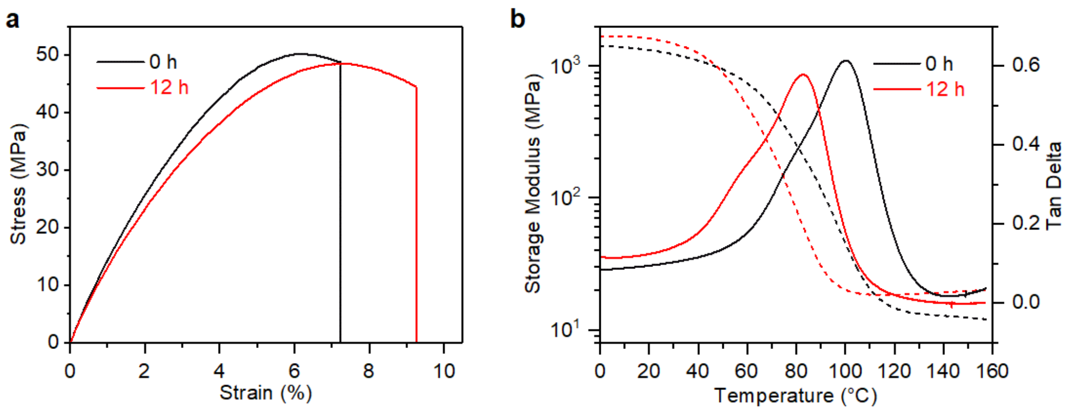

Supplementary Figure 14 Thermal and mechanical characterization of sample HUBM-co-HBA before and after topology isomerization. a Stress-strain curves. **b** DMA curves.

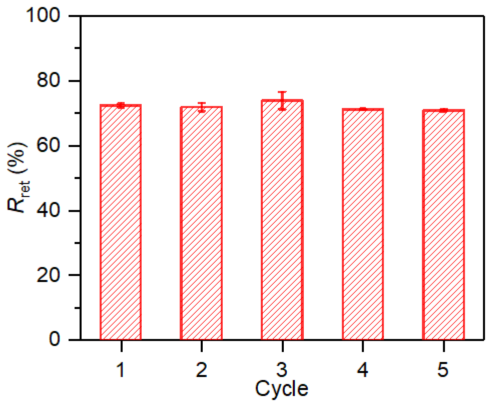

Supplementary Figure 15 Shape retention after multiple cycles of shape reconfiguration at 80 °C for HUBM-co-PPGA.

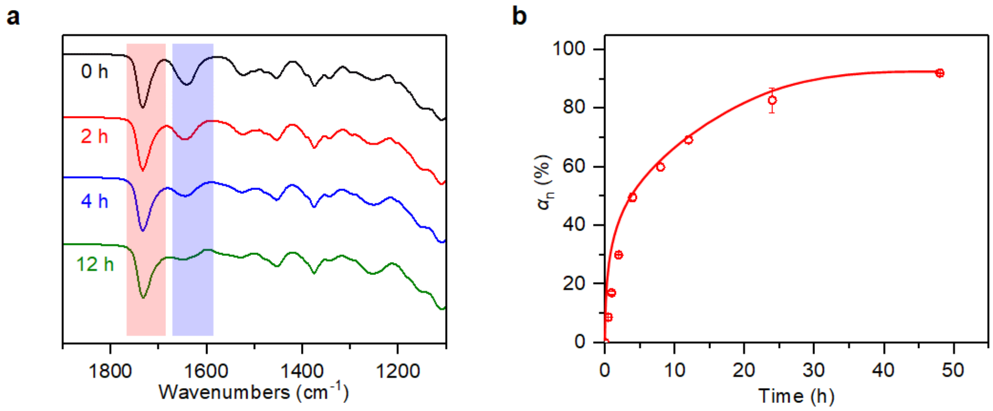

Supplementary Figure 16 Measurement of hindered urea conversion for sample HUBM-co-PPGA. a Change of the representative Fourier transform infrared (FTIR) spectra at 120 °C over time. **b** Time evolution of the hindered urea bond conversion α_n at 120 °C.

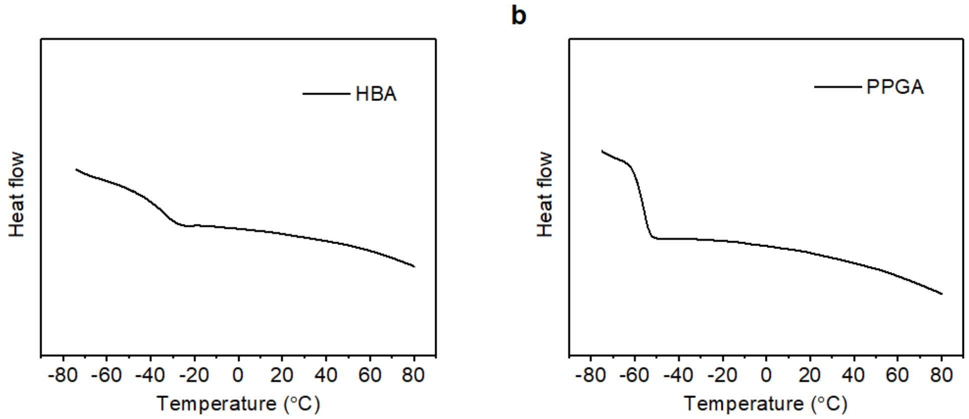

Supplementary Figure 17 DSC curves. a Homopolymer of HBA. **b** Homopolymer of PPGA. 1 wt% of PEGDA was employed as the photo-crosslinker for preparing the test samples. The glass transition temperatures for **a** and **b** were tested as -35 °C and -55 °C.

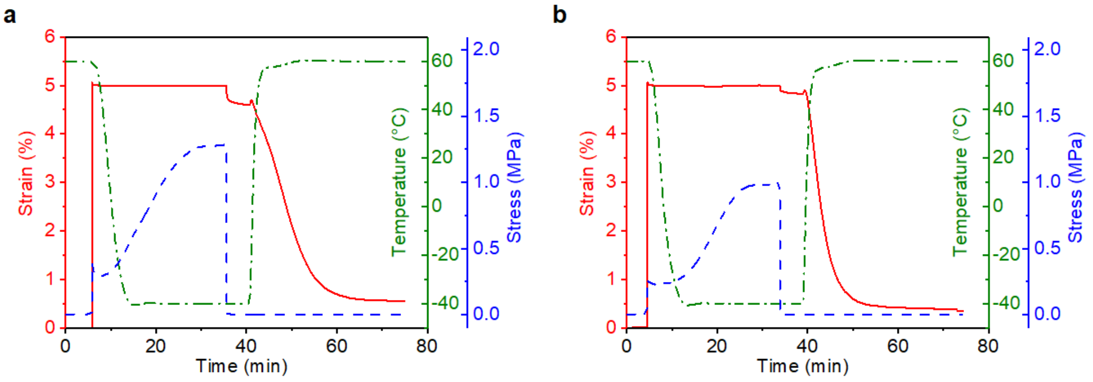

Supplementary Figure 18 Shape memory cycles of HUBM-co-PPGA. a Original sample. **b** Sample after annealing at 120 °C for 24 hours. The corresponding R_f and R_r values were calculated as 92% and 89% for **a**, and 97% and 93% for **b**.

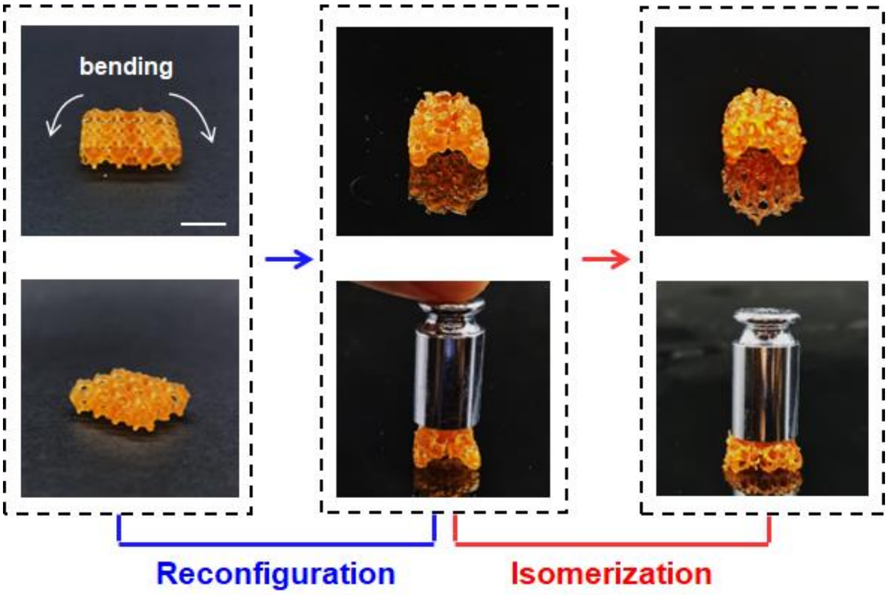

Supplementary Figure 19 Sequential reprogramming of the shape and mechanical properties. Scale bar, 10 mm.

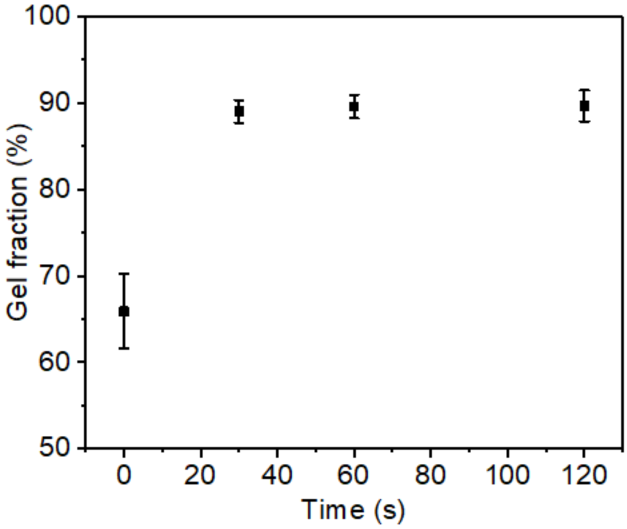

Supplementary Figure 20 The gel fraction of the printed sample (thickness: 0.4 mm) after different exposure times in a UV chamber. The results showed that the gel fractions reach an equilibrium state above 90% after being irradiated for 30 s. Thus, the post-curing can be regarded as finished with an exposure time of 120 s.

Supplementary Table 1 The swelling ratio Q, the volume fractions of the polymer V_K and the average molecular weight between crosslinks M_c of samples HUBM-co-PPGA upon annealing for different times.

Sample	0 h	2 h	4 h	24 h
Swelling ratio Q	0.75±0.07	0.90±0.04	0.99±0.01	1.24±0.03
Volume fractions of the polymer V_K	0.57±0.02	0.53±0.01	0.50±0.01	0.46±0.01
Average molecular weight between crosslinks M_c (kg/mol)	186±14	216±7	233±3	271±4

Supplementary Table 2 The gel fraction of samples HUBM-co-PPGA upon annealing for different times.

Sample	0 h	2 h	4 h	24 h
Gel fraction(%)	90.9±2.0	92.4±0.8	93.4±0.9	96.2±1.1

Supplementary Table 3 Mechanical properties of samples HUBM-co-PPGA via UV-curing or 3D printing.

Sample		0 h	2 h	4 h	24 h
UV-curing	Young's modulus (MPa)	70±5	43±3	32±1	7±1
	Strain-at-break (%)	22±2	26±2	39±8	55±6
3D printing	Young's modulus (MPa)	73±11	36±2	31±4	8±1
	Strain-at-break (%)	17±3	29±7	37±7	57±12

Supplementary Table 4 Mechanical properties of samples HUBM-co-PPGA after being stored at room temperature for 2 weeks.

Sample	0 h	2 h	4 h	24 h
Young's modulus (MPa)	71±5	34±3	28±3	6±1
Strain-at-break (%)	21±3	28±7	31±3	53±9

Supplementary References

1. Flory, P. J. Statistical mechanics of swelling of network structures. *J. Chem. Phys.* **18**, 108-111 (1950).

2. Orwoll, R.A. & Arnold, P.A. Polymer-solvent interaction parameter χ, in: J. Mark (Ed.), *Physical Properties of Polymers Handbook*, Springer, New York, 233-257 (2007).
